# T89. DEFINING COGNITIVE “NORMALITY” IN SCHIZOPHRENIA: PREVALENCE OF BROAD AND NARROW CRITERIA AND RELATION TO CLINICAL AND FUNCTIONAL STATUS

**DOI:** 10.1093/schbul/sby016.365

**Published:** 2018-04-01

**Authors:** Melissa Parlar, Walter Heinrichs, Stephanie McDermid Vaz, Clayton Wilson, Farena Pinnock

**Affiliations:** 1 York University; 2 McMaster University; 3 Ryerson University

## Abstract

**Background:**

Cognitive dysfunction is considered a core feature of schizophrenia. Nonetheless, patients with the illness overlap with healthy controls on many tasks, giving rise to the identification of subpopulations with relatively normal cognitive performance. However, the prevalence and implications of these subgroups for understanding schizophrenia are unclear because “normality” criteria vary. Estimates of the frequency of normal range performance in the patient population are as low as 0% and as high as 89%. This study examines the relation between different normality criteria and normality prevalence. It also assesses functional outcome and symptom severity in cognitively normal and impaired subgroups.

**Methods:**

“Narrow” (IQ) and “broad” (MATRICS Consensus Cognitive Battery; MCCB) cognitive normality criteria were applied to data from schizophrenia (n = 99) and healthy control samples (n = 80). Functional outcome was assessed with the Multidimensional Scale of Independent Functioning (MSIF). The Positive and Negative Syndrome Scale (PANSS) was administered to measure symptom severity.

**Results:**

Cognitive normality ranged from 13% (broad criterion) to 47% (narrow criterion) among patients. Patients meeting both broad (MCCB) and narrow (IQ) definitions were functionally disadvantaged compared to cognitively normal controls (t(63) = 7.05, p < .01; t(72) = 9.97, p < .01, respectively). However, cognitively normal patients showed no functional (MSIF) advantage relative to cognitively impaired patients based on both broad and narrow definitions of cognitive normality (t(95) = .43, p = .67; t(74) = -1.04, p = .30, respectively). Functioning did not differ between IQ and MCCB based cognitively normal patients (t(51) = .61, p = .55). Moreover, broad and narrow definitions of cognitive normality were not associated with differences in symptom severities relative to cognitively impaired patients. This held true for both positive (t(97) = 1.39, p = .17; t(76) = -.72, p = .47, broad and narrow definitions, respectively) and negative (t(97) = .98, p = .33; t(76) = -1.07, p = .29, broad and narrow definitions, respectively) symptom severity on the PANSS.

**Discussion:**

Our data show that the prevalence of cognitive performance normality varies widely with the breadth of the normality criterion. However, regardless of the criterion applied, cognitively normal patients remain functionally disadvantaged relative to cognitively normal controls. Perhaps more importantly, however defined, cognitively normal patients demonstrate no advantage in functionality relative to cognitively impaired patients. Thus, patients meeting the broad definition of cognitive normality are not functionally advantaged relative to those meeting the narrow definition. We also found that varying definitions of cognitive normality/impairment have no implications for the severity of psychotic psychopathology in treated outpatients. Overall, the current study suggests that the reported prevalence of cognitive normality in schizophrenia is largely a product of definitional approaches. At the same time, the data cast doubt on the functional importance of preserved and proficient cognition regardless of definition and suggest that cognitive normality does not confer an advantage in terms of reduced symptom severity.

